# Depressive symptoms and receipt of pensions: a cross-sectional analysis of the ELSI-Brazil study

**DOI:** 10.1590/S2237-96222023000300017.en

**Published:** 2023-12-01

**Authors:** Ana Paula Goulart de Freitas, Klaide Lopes de Sena, Jôsi Fernandes de Castro Rodrigues

**Affiliations:** 1 Faculdade de Ciências Médicas de Minas Gerais, Departamento de Pesquisa e Extensão, Belo Horizonte, MG, Brazil; 2 Faculdade de Ciências Médicas de Minas Gerais, Departamento de Saúde Coletiva e Preventiva, Belo Horizonte, MG, Brazil

**Keywords:** Retirement, Pensions, Depression, Elderly, Cross-sectional studies, Jubilación, Pensiones, Depresión, Anciano, Estudios Transversales, Aposentadoria, Pensões, Depressão, Idoso, Estudos Transversais

## Abstract

**Objective:**

To investigate association between depressive symptoms and receipt of retirement pensions or other pensions in the Brazilian population aged 50 years or older.

**Method:**

This was a cross-sectional study with participants from the baseline (2015-2016) of the Longitudinal Study of the Health of Elderly Brazilians. Depressive symptoms were measured by the eight-item Center for Epidemiologic Studies Depression Scale. Prevalence ratios (PR) were obtained by Poisson regression.

**Results:**

Among the total 8,469 participants, 33.9% (95%CI 32.8;34.9) reported depressive symptoms and 52.8% (95%CI 51.8;53.9) of the participants received a retirement or other pension. Prevalence of depressive symptoms was lower among participants receiving a retirement or other pension (PR = 0.79; 95%CI 0.73;0.86). Association remained significant after adjustments for sociodemographic and health indicators (PR = 0.84; 95%CI 0.76;0.92).

**Conclusion:**

Participants who receive retirement or other pensions are less likely to report depressive symptoms.

## Study contributions

**Main results:**

Prevalence of depressive symptoms was 33.9% in the population studied and, after adjusted analysis, it remained statistically lower among participants who received retirement or other pensions.

**Implications for services:**

Prevalence of depressive symptoms was higher in individuals who do not receive retirement or other pensions, which demands attention from health services in caring for this public in vulnerable situations regarding social security.

**Perspectives:**

Carrying out longitudinal studies capable of assessing the temporality of association between receiving retirement or other pensions and the mental health of the elderly, thus contributing to better knowledge about the social determinants of mental health.

## RESUMEN

**Objetivo:**

Investigar la asociación entre los síntomas depresivos y la percepción de jubilaciones o pensiones en la población brasileña de 50 años o más.

**Método:**

Estudio transversal con participantes del Estudio Longitudinal de la Salud del Anciano Brasileño (2015-2016). Los síntomas depresivos se midieron mediante la escala The eight-item Center for Epidemiologic Studies Depression. Las razones de prevalencia (RP) se obtuvieron por regresión de Poisson.

**Resultados:**

La prevalencia de síntomas depresivos fue del 33,9% (IC_95_% 32,8;34,9) y 52,8% (IC_95_% 51,8;53,9) de los participantes recibían jubilación o pensión. La prevalencia de síntomas depresivos fue menor entre los participantes que recibían jubilación o pensión (PR = 0,79; IC_95_% 0,73;0,86). La asociación siguió siendo significativa tras ajustarse por indicadores sociodemográficos y de salud (PR = 0,84; IC_95_% 0,76;0,92).

**Conclusión:**

Los participantes que reciben una jubilación o pensión tienen menos probabilidades de manifestar síntomas depresivos.

## RESUMO

**Objetivo:**

Investigar a associação entre recebimento de aposentadorias ou pensões e sintomas depressivos na população brasileira com 50 anos ou mais.

**Método:**

Estudo transversal com participantes da linha de base (2015-2016) do Estudo Longitudinal da Saúde dos Idosos Brasileiros. Sintomas depressivos foram aferidos pela escala *The eight-item Center for Epidemiologic Studies Depression*. Razões de prevalência (RP) foram obtidas pela regressão de Poisson.

**Resultados:**

Dos 8.469 participantes, 33,9% (IC_95%_ 32,8;34,9) relataram sintomas depressivos e 52,8% (IC_95%_ 51,8;53,9) recebiam aposentadoria ou pensão. A prevalência de sintomas depressivos foi menor entre os participantes que recebiam aposentadoria ou pensão (RP = 0,79; IC_95%_ 0,73;0,86). A associação permaneceu significativa após ajustes por indicadores sociodemográficos e de saúde (RP = 0,84; IC_95%_ 0,76;0,92).

**Conclusão:**

Participantes que recebiam aposentadoria ou pensão são menos propensos a relatarem sintomas depressivos.

## INTRODUCTION

The process of population aging in Brazil demonstrates an accelerated trend of evolution.^1^ This phenomenon is the result of the concomitant reduction in fertility rates and the increase in the population’s life expectancy, which culminate in significant changes in the country’s age structure.^2^ In this context, it is expected that a significant portion of the population will go through the retirement process. This transition entails considerable transformations in multiple aspects of life, encompassing social, economic and individual factors.^3,4^

Studies exploring the relationship between retirement and depression have found inconsistent results.^5^ However, a study conducted in China showed that receiving a retirement pension or other pension can reduce the prevalence of depression in the elderly.^6^ Additionally, it is possible to interpret the benefit as a supplement to income, playing a fundamental role in preserving the mental health of this population, by ensuring their ability to acquire and use resources aimed at promoting health.^7^

Some studies show that receiving retirement or other pensions plays a significant role in improving depressive symptoms.^7,8^ Presence of depressive symptoms has been correlated with characteristics such being female, low schooling, not having a partner and unfavorable self-rated health.^9^

Analysis of the impacts of retirement on mental health remains little explored, particularly in the Brazilian context. This is a complex scenario, as it is difficult to establish a clear causal relationship between these two elements, as a series of factors influence the decision to retire. This occurs due to the influence of certain important aspects, such as age, work environment characteristics, nature of professional activities and underlying health conditions. It should be highlighted that what determines the retirement process is not external to or independent of health-related aspects.^10^

The rapid growth of the retired population in a middle-income country is evident. This results in a gap in guaranteeing the resources necessary to promote health and well-being.^11^ The scenario of a low socioeconomic level together with prolonged life expectancy after retirement can generate psychological stress.^12^ This phenomenon is important, since changes in the mental health of individuals impact the use of health services.^13^ Therefore, studies that address this topic are relevant for the development of public policies aimed at more targeted and effective health care.^13^

Due to the increase in the elderly population in Brazil and, consequently, in the number of retirees as well, this topic has acquired relevance. This occurs because depressive symptoms have a significant impact on both the assessment of mental well-being and also the quality of life of this population. Therefore, this article sought to investigate association between depressive symptoms and receiving retirement or other pensions in the Brazilian population aged 50 or over. 

## METHODS

We conducted a cross-sectional study using baseline data from the Longitudinal Study of the Health of Elderly Brazilians (*Estudo Longitudinal da Saúde dos Idosos Brasileiros - ELSI-Brasil*, 2015-2016), a prospective household-based cohort study, with the main objective of investigating the dynamics of the aging of the Brazilian population and its determinants. The sample was designed to be representative of the non-institutionalized Brazilian population aged 50 or over.^14^ The baseline survey included 9,412 participants living in 70 municipalities in different regions of Brazil.^14^ Participants were selected based on selection stages that considered the municipality, census tract and household. Further details about the study design, selection criteria, quality assurance and control, as well as cohort characteristics, have been reported elsewhere.^14^

The following variables were assessed:

a) Sociodemographic variables:
  - Sex (male and female);
  - Age group (50-59, 60-69, 70-79, 80 years or over);
  - Per capita monthly household income (categorized into quintiles, representing income distribution from lowest to highest, with the first quintile referring to the lowest income range, and the fifth quintile corresponding to the highest income range);
  - Complete years of schooling (less than 4 years, 4-7 years, 8 years or more);
  - Current marital status (married/common law marriage, separated/divorced, single, widowed).
b) Self-rated health (very good or excellent, good, regular, poor, very poor).
c) Depressive symptoms.
d) Receipt of retirement pension or other pension.

The dependent variable, depressive symptoms, was obtained using the Eight-item Center for Epidemiologic Studies Depression Scale (CES-D8),^15^ which consists of a questionnaire with eight questions. This was categorized into presence of depressive symptoms when the scale score was equal to or greater than 4 points, and absence of depressive symptoms if the scale score was less than 4 points. To assign numerical values, the value “1” was assigned to responses indicative of depressive symptoms on the CES-D8 Scale, while the value “0” was assigned to negative responses. The cutoff point used was specified by Van de Velde, Levecque and Bracke (2009) and McGovern and Nazroo (2015).^16,17^

The main independent variable was the receipt of a retirement pension or other pension, which was measured using the following question: *Do you receive a retirement pension or other pension from the federal Social Security Institute (INSS) or from the federal, state, municipal government or private retirement pension fund?* The answer options were “yes” or “no”.

We performed a descriptive analysis of the sociodemographic characteristics of the study participants, and prevalence of the presence of depressive symptoms was estimated according to the explanatory variable. Continuous variables were expressed as means and standard deviations (SD); the categorical variables were expressed in percentages. 

Association between presence of depressive symptoms and receiving a retirement pension or other pension was assessed using crude and adjusted prevalence ratios (PR), together with 95% confidence intervals (95%CI). These estimates were obtained using the Poisson regression model, and statistical significance was assessed using the p-value derived from the Wald test. In the adjusted model, all independent variables were simultaneously incorporated. Additionally, a similar analysis was conducted stratifying by sex. 

For all analyses, the weightings of individuals and sampling parameters were taken into account using procedures for complex samples with Stata 14.0 software, in the survey function.

The ELSI-Brazil study was approved by the Research Ethics Committee (Certificate of Submission for Ethical Appraisal: 34649814.3.0000.5091, Opinion No. 2.111.911, issued on June 9, 2017). All individuals were informed in detail about the objectives and procedures of the study and, after reading and understanding the information, they signed a Free and Informed Consent Form.

## RESULTS

**Figure 1:**
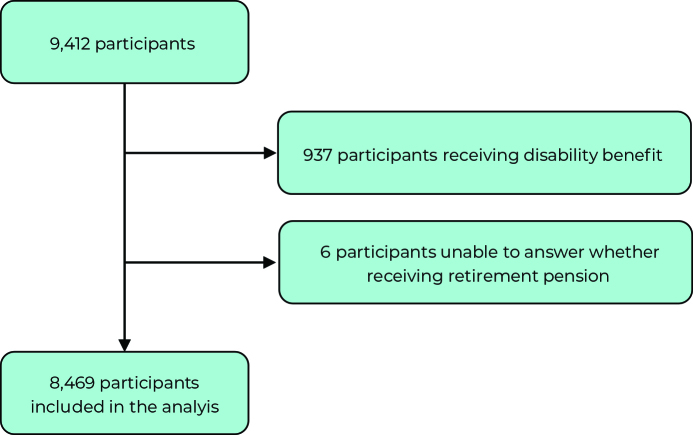
Final composition of the study sample following application of the inclusion criteria, Longitudinal Study of the Health of Elderly Brazilians (ELSI-Brazil), 2015-2016

This investigation excluded participants who retired due to disability (n = 937) and those who were unable to provide information about receiving a retirement pension (n = 6). Thus, the sample to be analyzed comprised 8,469 (89.98%) of the total interviewees (Figure 1). The exclusion of participants retired due to disability was based on the fact that mental and behavioral disorders are the main cause of retirement for this reason.

Of the 8,469 individuals included in the descriptive analysis, the majority were female (58.2%) and reported their marital status as being married or in a common law marriage (57.8%). Average age was 69.4 years (SD = 9.3 years). The most frequent age group was between 50-59 years (43%). Furthermore, the majority stated that they had less than four years of schooling (36.4%), followed by eight years or more (33.4%). With regard to *per capita* household income, the majority of participants were in the first quintile (21.5%). Self-rated health was perceived as good by 37.5% (Table 1).

**Table 1:** Study participant (≥ 50 years old) characteristics, according to receipt of retirement pension or other pension, Longitudinal Study of the Health of Elderly Brazilians (ELSI-Brazil), 2015-2016

Variables	Receipt of retirement pension or other pension		
	Total (n = 8,469)%	No (n = 3,990)%	Yes (n = 4,479)%
**Sex**			
Female	58.2	53.5	62.3
Male	41.8	46.5	37.7
**Age (years)**			
50-59	43.0	74.5	15.0
60-69	29.8	21.0	37.6
70-79	19.0	3.5	32.7
80 or over	8.2	1.0	14.7
**Schooling (years)**			
< 4	36.4	27.4	44.4
4-7	30.2	33.4	27.3
8 or more	33.4	39.2	28.3
***Per capita* household income (quintile)**			
1^st^ (lowest)	21.5	29.7	14.1
2^nd^	19.2	22.6	16.2
3^rd^	19.3	14.2	23.9
4^th^	19.6	18.4	20.7
5^th^ (highest)	20.4	15.1	25.1
**Marital status**			
Married/common law marriage	57.8	67.6	49.1
Separated. divorced	11.9	14.3	9.7
Single	11.0	12.8	9.4
**Self-rated health**			
Very good or excellent	6.7	7.0	6.3
Good	37.5	35.9	38.9
Regular	44.7	45.5	44.0
Poor	7.2	7.4	7.2
Very poor	3.9	4.2	3.6

The majority (62.3%) of the participants who declared receiving a retirement or other pension were female. The 60-69 age group was predominant (37.6%). Around 44% declared having less than four years of schooling and 25.1% had *per capita* household income in the highest quintile. Regarding marital status, 49.1% of those who received a retirement or other pension were married or living in a common law marriage. Furthermore, 44% reported regular self-rated health (Table 1). Prevalence of depressive symptoms among participants in this study was 33.8% (95%CI 32.8;34.9).

Table 2 presents the crude and adjusted analyses of the association of the characteristics studied and of depressive symptoms. In the crude analysis, we found that prevalence of depressive symptoms was 45% lower (PR = 0.55; 95%CI 0.51;0.59) in females compared to males. Furthermore, there was a lower prevalence of depressive symptoms among those who received a retirement or other pension, particularly in individuals in the 70-79 age group (PR = 0.77; 95%CI 0.67;0.88), those with over eight years of schooling (PR = 0.68; 95%CI 0.60;0.78), *per capita* household income in the fifth quintile (PR = 0.51; 95%CI 0.45;0.59) and those who were divorced (PR = 0.75; 95%CI 0.66;0.85). Prevalence of depressive symptoms was significantly higher (PR = 5.89; 95%CI 4.36;7.96) for participants with very poor self-rated health.

**Table 2:** Crude and adjusted prevalence ratios (PR) of characteristics associated with depressive symptoms (n =7,473), Longitudinal Study of the Health of Elderly Brazilians (ELSI-Brazil), 2015-2016

Variables	Not adjusted			Adjusted	
	PR (95%CI^a^)	p-value ^b^		PR (95%CI^a^)	p-value ^b^
**Receipt of retirement pension or other pension**		**< 0.001**			**< 0.001**
No	1.00			1.00	
Yes	0.79 (0.73;0.86)			0.84 (0.76;0.92)	
**Sex**		**< 0.001**			**< 0.001**
Male	1.00			1.00	
Female	0.55 (0.51;0.59)			0.60 (0.56;0.66)	
**Age group (years)**		**< 0.001**			**0.011**
50-59	1.00			1.00	
60-69	0.86 (0.78;0.95)			0.89 (0.82;0.97)	
70-79	0.77 (0.67;0.88)			0.77 (0.68;0.89)	
80 or over	0.94 (0.81;1.09)			0.89 (0.76;1.05)	
**Schooling (years)**		**< 0.001**			**0.026**
< 4	1.00			1.00	
4-7	0.92 (0.82;1.02)			0.96 (0.88;1.04)	
8 or more	0.68 (0.60;0.78)			0.86 (0.77;0.96)	
***Per capita* household income (quintile)**		**< 0.001**			**0.009**
1^st^ (lowest)	1.00			1.00	
2^nd^	0.89 (0.80;0.98)			0.98 (0.88;1.08)	
3^rd^	0.80 (0.72;0.90)			0.97 (0.87;1.09)	
4^th^	0.71 (0.63;0.80)			0.90 (0.80;1.02)	
5^th^ (highest)	0.51 (0.45;0.59)			0.80 (0.70;0.90)	
**Marital status**		**< 0.001**			**< 0.001**
Married/common law marriage	1.00			1.00	
Separated, divorced	0.75 (0.66;0.85)			0.84 (0.75;0.96)	
Single	1.06 (0.91;1.24)			1.11 (0.96;1.28)	
Widowed	1.04 (0.91;1.19)			1.15 (1.00;1.33)	
**Self-rated health**		**< 0.001**			**< 0.001**
Very good or excellent	1.00			1.00	
Good	1.63 (1.21;2.18)			1.65 (1.21;2.25)	
Regular	2.91 (2.15;3.93)			2.82 (2.05;3.87)	
Poor	4.81 (3.56;6.49)			4.40 (3.21;6.04)	
Very poor	5.89 (4.36;7.96)			5.04 (3.63;7.00)	

a) 95%CI: 95% confidence interval; b) Wald test; p-value < 0.05 in bold type.

The adjusted analysis corroborated the findings of the crude analysis. Prevalence of depressive symptoms was 16% lower among participants who received a retirement or other pension (PR = 0.84; 95%CI 0.76;0.92) compared to those who did not. Furthermore, depressive symptoms were 40% less common in females compared to males (PR = 0.60; 95%CI 0.56;0.66). We also found that these symptoms were 23% less frequent in participants aged between 70 and 79 years, compared to those between 50 and 59 years old (PR = 0.77; 95%CI 0.68;0.89). In addition, prevalence was 14% lower among those who had more than eight years of schooling compared to those with less than four years (PR = 0.86; 95%CI 0.77;0.96). Among participants falling into the fifth quintile of *per capita* household income, prevalence of depressive symptoms was 20% lower compared to those in the lowest income quintile (PR = 0.80; 95%CI 0.70;0.90). Depressive symptoms were 16% less frequent among divorced people compared to those who were married or in a common law marriage (PR = 0.84; 95%CI 0.75;0.96).

A suggestion of positive dose-response slope was found between depressive symptoms and self-rated health, whereby prevalence was 400.4% higher among individuals who self-rated their health as very poor (PR = 5.04; 95%CI 3.63;7.00).

When stratifying the adjusted analysis by sex, the results differed from those found for the total sample (Table 3). Furthermore, in the case of females, inverse associations persisted between the presence of depressive symptoms and variables such as receipt of a retirement pension, the 60-69 and 70-79 age groups, eight or more years of schooling and falling into the highest income quintile. In the case of males, associations only remained with the highest income quintile and being separated/divorced. However, being widowed was positively associated with the presence of depressive symptoms among males. In both sexes, regular, poor or very poor self-rated health remained positively associated with the presence of depressive symptoms, although with a smaller magnitude among males.

**Table 3:** Adjusted prevalence ratios (PR) of characteristics associated with depressive symptoms, by sex (n =7,473), Longitudinal Study of the Health of Elderly Brazilians (ELSI-Brazil), 2015-2016

Variables	Female	Male
	PR (95%CI^a^)	PR (95%CI^a^)
**Receipt of retirement pension or other pension**		
No	1.00	1.00
Yes	0.86 (0.78;0.95)	0.86 (0.71;1.03)
**Age group (years)**		
50-59	1.00	1.00
60-69	0.89 (0.81;0.98)	0.88 (0.74;1.05)
70-79	0.74 (0.63;0.86)	0.85 (0.64;1.12)
80 or over	0.90 (0.77;1.05)	0.88 (0.61;1.28)
**Schooling (years)**		
< 4	1.00	1.00
4-7	0.94 (0.85;1.04)	1.00 (0.85;1.19)
8 or more	0.87 (0.77;0.98)	0.85 (0.69;1.06)
***Per capita* household income (quintile)**		
1^st^ (lowest)	1.00	1.00
2^nd^	0.96 (0.85;1.07)	0.99 (0.78;1.24)
3^rd^	1.00 (0.87;1.14)	0.88 (0.70;1.10)
4^th^	0.94 (0.83;1.06)	0.81 (0.65;1.01)
5^th^ (highest)	0.82 (0.72;0.95)	0.72 (0.53;0.97)
**Marital status**		
Married/common law marriage	1.00	1.00
Separated, divorced	0.94 (0.82;1.07)	0.66 (0.51;0.87)
Single	1.15 (0.99;1.33)	1.00 (0.72;1.38)
Widowed	1.13 (0.97;1.32)	1.48 (1.07;2.05)
**Self-rated health**		
Very good or excellent	1.00	1.00
Good	2.03 (1.39;2.96)	1.09 (0.65;1.83)
Regular	3.25 (2.21;4.80)	2.16 (1.28;3.64)
Poor	4.66 (3.20;6.79)	4.08 (2.38;6.99)
Very poor	5.47 (3.75;7.97)	4.77 (2.73;8.32)

a) 95%CI: 95% confidence interval.

## DISCUSSION

This study showed that approximately one third of non-institutionalized Brazilians, aged 50 or over reported presence of depressive symptoms. Presence of these symptoms was lower among participants who received a retirement or other pension, even after adjustments for the covariables analyzed. The results obtained corroborate the established hypothesis, demonstrating that prevalence of depressive symptoms was lower among those receiving a retirement pension, particularly among individuals in older age groups, with higher educational levels, higher family income, as well as among those were married and had good self-rated health.

A study conducted in 2022, using data from the China Family Panel Studies for the years 2012, 2016 and 2018, with 2,157 elderly people living in rural areas of China, found that the rural pension system had a significant effect on reducing mental health problems and medical costs associated with depression.^8^ Another study, based on data from the 2014 Chinese Longitudinal Aging Social Survey, involving individuals aged 60 or over, revealed significant negative association between the granting of pensions and the manifestation of depressive symptoms.^7^ This beneficial effect can be attributed to the increase in regular income and the reduction of economic uncertainty.^7^ Additionally, both studies found that prevalence of depressive symptoms among retirement pension beneficiaries is lower when compared to those without this benefit.^7,8^

These results are consistent with previous studies, which indicate a positive effect of retirement on people’s health.^9^ In 2015, a study involving 2,000 Chinese citizens over the age of 40 identified significant association between the lack of financial support during retirement and an increased chance of compromised mental health.^12^ The analyses performed in our study corroborate the findings, indicating lower prevalence of depressive symptoms among retirement or other pension beneficiaries in Brazil. Congruently, a cross-sectional study conducted in China, encompassing 9,672 individuals over 60 years of age, covering data collected between 2012 and 2018, revealed that the presence of depressive symptoms was significantly lower among pensioners.^19^ This finding reinforces the results discussed in the article, highlighting that retirees who receive a retirement or other pension are less likely to report depressive symptoms. 

Lower prevalence of depressive symptoms was identified in females compared to males, after retirement. In line with this, a review the objective of which was to synthesize the literature on risk factors for the development of depression during the pandemic, highlighted that female pensioners or those financially prepared for retirement had better mental health.^20^ Furthermore, a study that used data from the 2010 and 2012 China Family Panel Studies, involving individuals aged 45 years or over, found that those with social security insurance tended to report more robust mental health compared to those who were not insured, however, significant differences between the sexes were not found.^21^

The results of this study indicated that, even considering the covariables, prevalence of depressive symptoms was lower among people with higher levels of education. Two studies address this topic. The first took place between 2009 and 2010, in Florianópolis, with individuals over 60 years old.^23^ The second used data from the Health and Retirement Study, conducted between 2012 and 2013, with samples from China, England, Mexico and the United States, focused on retirees.^22^ Both studies reported that people with lower levels of education had poorer self-rated health and greater mental health impairment.^22,23^ This suggests that the level of education received by a person over their life directly or indirectly influences socioeconomic determinants that negatively impact quality of life, such as the emergence of depressive symptoms during retirement.

According to data collected between 2012 and 2013 by the Health and Retirement Study, prevalence of depressive symptoms was higher in Mexico (7.16%) and China (7%), when compared to the United States (4.21%) and England (3.98%), in addition to being more prevalent in individuals with an unfavorable socioeconomic situation.^22^ Studies indicate greater economic security for those who receive pensions, at the same time that depressive symptoms are more prevalent among those with unfavorable socioeconomic conditions.^18,24-26^ In our study, the association of the highest *per capita* household income quintile with a lower prevalence of depressive symptoms reinforces these findings. 

Our study identified greater prevalence of depressive symptoms among males who were widowers. A study carried out between 2014 and 2019 with 6,281 individuals aged 40 or over, living in a rural community in South Africa, also revealed a significant association between being a widower and having a greater number of depressive symptoms.^27^ Similarly, a study carried out between 2015 and 2016, involving individuals aged 60 or over in the municipality of Cajazeiras, found positive association between being widowed and depressive symptoms, although it did not perform analysis stratified by sex.^28^ Furthermore, we found lower prevalence of depressive symptoms among separated/divorced males, unlike other studies.^23,27,28^ It is essential to consider the interaction of factors, such as mental health history, social support, individual circumstances, which can influence in a variable manner the response to divorce and its effects on mental health over time.^23,27,28^ Therefore, research in this field is essential for a more comprehensive and accurate understanding of the psychological implications of divorce, particularly in relation to older age groups.

Furthermore, we found positive association between presence of depressive symptoms and poor or very poor self-rated health. This finding is in line with the results of other studies, since presence of depressive symptoms has been correlated with negative perception of one’s own health.^20,24^

This study has limitations that need to be considered when interpreting the results. We emphasize the possibility of reverse causality bias. The study’s cross-sectional design makes it impossible to explore a causal relationship or the dynamics of how receiving a retirement pension or other pension is related to the mental health of the elderly. Additionally, this design favors the occurrence of survival bias, which may result in associations being overestimated.

Obtaining empirical evidence is essential for establishing a possible causal relationship between receiving a retirement pension or other pension and the mental health of the elderly. This effort can contribute to advancing knowledge about the social determinants of mental health in this age group. Furthermore, it is important to identify how receiving a retirement pension or other pension can play a protective role for the mental health of older people. Such discoveries have the potential to provide information for the formulation of public policies aimed at improving the quality of life of this population.

The results of longitudinal studies can inform policy decisions related to retirement and pensions. This highlights the importance of establishing an adequate and accessible pension system, considering the direct impact on the mental health of the elderly. It is important to highlight that the CES-D8 scale does not provide a diagnosis of depression, but rather a measure of depressive symptoms, in addition to resulting in different interpretations according to each individual’s understanding of the questions.

The strengths of this study include the large population base of the ELSI-Brazil cohort, as well as the inclusion of a representative sample of Brazilian adults aged 50 years and over. Furthermore, excluding those who are retirees due to health reasons constitutes a prudent approach, as this form of retirement is intrinsically influenced and can cause bias in the results when the outcome in question is the manifestation of depressive symptoms.

Finally, we conclude that depressive symptoms showed significant prevalence in the population studied, reinforcing its relevance as a public health problem. The results showed that, in addition to schooling and income, receiving a retirement pension and being divorced are associated with lower prevalence of these symptoms among older adults. On the other hand, certain characteristics were associated with higher prevalence of depressive symptoms: poor self-rated health and males who were widowers.

As such, this study highlights the need for intersectoral interventions that promote mental health before and during retirement. These characteristics should be considered in strategies to improve health standards in the transition to retirement, thus contributing to a more positive standard of well-being at this stage of life.
